# Reliability of shear-wave elastography in assessing thoracolumbar fascia elasticity in healthy male

**DOI:** 10.1038/s41598-020-77123-w

**Published:** 2020-11-17

**Authors:** Baizhen Chen, Hongzhou Zhao, Linrong Liao, Zhijie Zhang, Chunlong Liu

**Affiliations:** 1 Clinical College of Acupuncture, Moxibustion and Rehabilitation, Guangzhou University of Chinese Medicine, Guangzhou, China; 2 Department of Orthopedics and Traumatology of Integrated Chinese and Western Medicine Reduction Room, Tianjin Hospital, Tianjin, China; 3 Department of Rehabilitation, Jiangsu Provincial Yixing Jiuru Rehabilitation Hospital, Yixing, China; 4 Luoyang Orthopedic Hospital of Henan Province, Orthopedic Hospital of Henan Province, Luoyang, China

**Keywords:** Anatomy, Health care, Pathogenesis

## Abstract

The objectives of this study were to examine the intra and inter-operator reliability of shear wave elastography (SWE) device in quantifying the shear modulus of thoracolumbar fascia (TLF) and the device’s abilities to examine the shear modulus of the TLF during upper body forward. Twenty healthy male subjects participated in this study (mean age: 18.4 ± 0.7 years). Two independent operators performed the shear modulus of TLF during upper body forward using SWE, and interclass correlation coefficient (ICC) and minimum detectable change (MDC) were calculated. The shear modulus of the TLF was quantified by operator A using SWE at upper body forward 60°. The intra-operator (ICC = 0.860–0.938) and inter-operator (ICC = 0.904–0.944) reliabilities for measuring the shear modulus of the TLF with the upper body forward 0° were rated as both excellent, and the MDC was 4.71 kPa. The TLF shear modulus of upper body forward 60°was increased 45.5% (L3) and 55.0% (L4) than that of upper body forward 0°. The results indicate that the SWE is a dependable tool to quantify the shear modulus of TLF and monitor its dynamic changes. Therefore, this device can be used for biomechanical study and intervention experiments of TLF.

## Introduction

The thoracolumbar fascia (TLF), as a support band or collateral ligament, is mainly responsible for transmitting and absorbing loads in twisting trunk and maintaining body posture. They play an important role in maintaining spinal stability and transmitting^1–3^. Studies have demonstrated that the increase of thoracolumbar fascia (TLF) hardness is associated with low back pain (LBP)^4,5^. However, the elastic properties of TLF lack specific vivo numerical data. Thus, the detection methods to quantify the elastic properties of TLF in a quick and reliable manner may provide useful information for the biomechanical study of spine and the clinical research of LBP and fascia therapy.

Few techniques already exist to assess the vivo stiffness of soft tissue, as MyotonPRO and magnetic resonance elastography (MRE)^6,7^. However, the MyotonPRO cannot provide images of the measurement area. The MRE needs to restrict the subject's position during measurement^8^. Therefore, none of these techniques meet the requirements of quantitative and dynamic monitoring of TLF elastic performance.

Shear wave elastography is a non-invasive imaging technique. It can quantitative measurement of local tissues elasticity in real time without restricting the patient position^9–11^. Our previous studies have shown that SWE is a reliable and effective tool for measuring the elastic properties of skeletal muscles, such as quadriceps muscle, gastrocnemius and upper trapezius and describing the dynamic biomechanical properties of the soft tissues^12–14^. However, SWE has not been applied to the elastic properties measurement of TLF. Furthermore, in clinical treatment and research, in order to reflect the progress of the disease, treatment effect and the accuracy of the test, patients usually must undergo multiple evaluations by one or more testers (doctors, therapists, researchers). Therefore, it is important to evaluate the intra- and inter-operator reliabilities of SWE. To our knowledge, intra- and inter-operator reliabilities of the SWE for the measurement of TLF shear modulus have not been determined. To collect reliable data in the future, it is necessary to determine the precision of SWE measurement, intra- and inter-operator reliabilities.

The objectives of this study are to determine the intra and inter-operator reliability of quantifying the TLF shear modulus using SWE techniques and to calculate the minimum detectable change (MDC) and investigate the change of elastic properties of TLF during various body positions.

## Methods

### Ethical approval

This study received institutional approval by the Human Subjects Ethics committee of Luoyang Orthopaedic Hospital of Henan Province (Number: KY2019-001-01).This research follows the principles of the Helsinki Declaration. Before the commencement of the study, all the subjects fully understood the purpose, process and safety of SWE and the basic rights of the subjects and signed the informed consent.

### Subjects

From September to November 2019, twenty healthy young male subjects were recruited from the Guangzhou University of Traditional Chinese Medicine in the study (mean age: 18.4 ± 0.7 years; mean height: 1.73 ± 0.05 m; mean weight: 61.1 ± 9.6 kg; mean body mass index: 20.5 ± 3.0 kg/m^2^). The subjects had no lower back pain or history of lumbar surgery or trauma for at least the previous 6 months, and everyone was right-handed. All the participants were banned from physical activity 48 h before the experiment.

### Equipment

The TLF shear modulus of the ultrasound examinations was performed using an Aixplorer ultrasound device(Aixplorer Supersonic Imagine, France) with a 40 mm linear array sensor(SL10-2, Supersonic Imagine, France), using the instrument’s default standard musculoskeletal settings, with the selection of SWE mode (enhanced mode, 85% opacity).The range of measurable was adjusted from 0 to 300 kPa.

### Procedures

**Figure 1:**
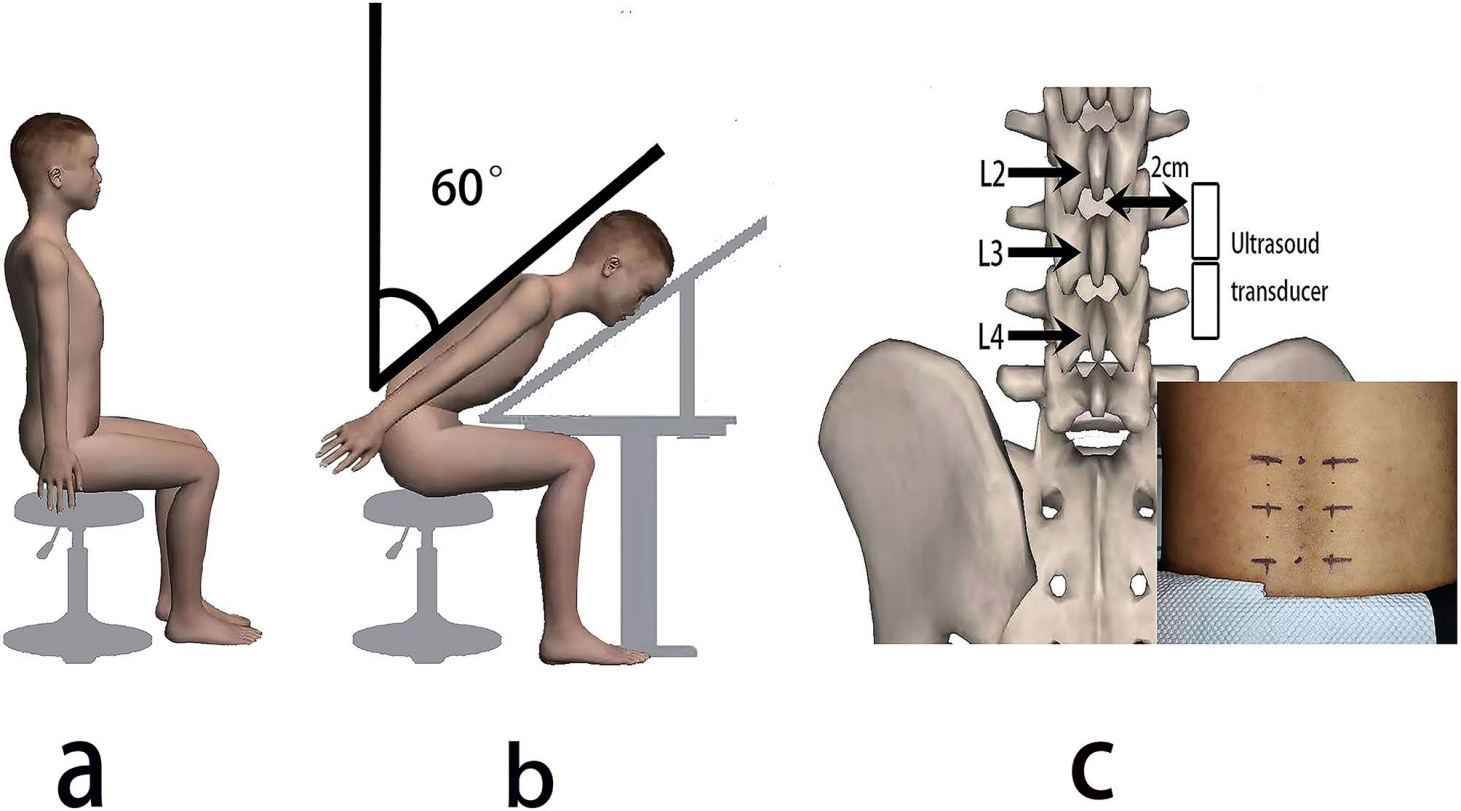
Illustration of the two postures (**a**) sitting; (**b**) sitting-forward 60°, and setting out plan of ultrasound transducer (**c**).

The TLF shear modulus was measured at the third and fourth lumbar vertebra levels (L3 and L4).The location of the L4 spinous process was identified by a body surface marker and reconfirmed by B-mode ultrasound. Then, the position of the L3 spines is determined by B-mode ultrasound. The longitudinal center of the probe at horizontally 2 cm from the right side of the L2–3 and the L3–4 midline (Fig. 1)^5,15,16^. These gauge points were marked with an oil-based pen. The marker of each experiment was cleaned at the end of the experiment.

All subjects performed experiments in a given posture: sitting, sitting-forward 60° (Fig. 1). In all postures, subjects are required to keep their heads in a neutral posturewith their upper limbs at their sides. In sitting posture, the subjects are required to keep their feet shoulder-width apart and both feet flat on the floor. Their legs are perpendicular to the ground, and their knees bent at 90°.We used a self-made sloping panel to keep the subjects' upper body at 0° and 60° of forward tilt.

**Figure 2:**
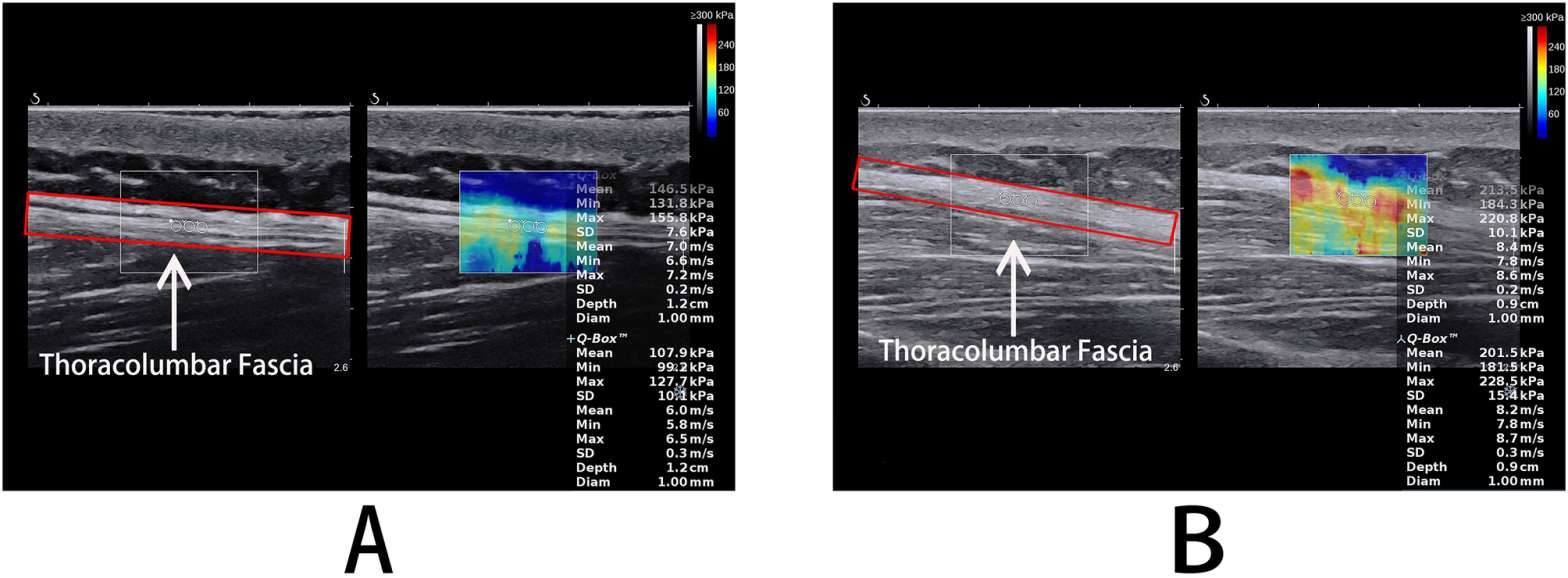
SWE maps of the thoracolumbar fascia. (**A**) sitting posture; (**B**) sitting-forward 60°.

To ensure that the muscles and fascia are at rest, the subjects were allowed to have a 5 min rest before testing. Then, the subjects were placed at appropriate posture, and ultrasonic gel was applied to the skin around the marked location. Under the B-mode image, the probe was placed perpendicular to the skin and slightly adjusted parallel to the upper tendon muscle fibers to obtain a clear image. Once the probe orientation was aligned with the direction of the muscle fibers, we switched to E mode to quantify the shear modulus of the upper TLF (Fig. 2). The size of the region of interest (ROIs) is set to match the thickness of the TLF shallow (a variable range is chosen to achieve a larger area and to keep the border of the ROIs at a certain distance from the upper subcutaneous tissue and the lower muscles^17^).Three measurements were taken for each measurement point, and the measurements were averaged. In addition, to avoid the influence of abdominal fluctuation caused by breathing, we specifically save the image at the end of expiration to ensure the consistency of test results.

For the intra-operator reliability, all subjects' right side TLF was examined by operator A according to the aforementioned program. Five days after the first measurement, operator A had made a second measurement of the same subject. To evaluate inter-operator reliability, all subjects' right side TLF were examined by operator A and operator B respectively, with the two measurements were taken 30 min apart. The measurement results were recorded by researcher C after all the tests were completed.

### Data analysis

**Figure 3:**
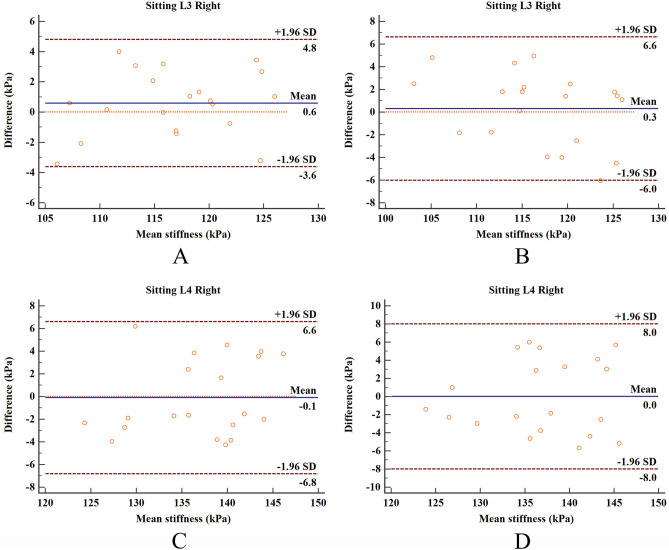
Bland and Altman plots of intra- and inter-operator reliabilities of TLF shear modulus. The difference in TLF stiffness between day 1 and day 5 is plotted against mean TLF stiffness (average of the 2 days for operator A) for each participant in the L3 (**A**) and L4 (**C**). The difference in TLF stiffness between operator A and operator B is plotted against mean TLF stiffness (average of the 2 operators) for each participant in the L3 (**B**) and L4 (**D**). In each picture, the continuous line is the mean difference and the dotted lines represent two SD above and below the mean difference.

SPSS 21.0 software (version 21.0 Chicago, IL) was used for statistical analysis. Demographic information was calculated by descriptive statistics, and measurement data was expressed as mean ± standard deviation (SD). For reliability analysis, an interclass correlation coefficient (ICC) with a 95% confidence interval was used as the reliability index within and between operators. ICC (3,1) (two-way mixed-effect model, consistency) and ICC (2,2) (two-way random effects model, absolute agreement) were used to assess reliability within-operator, between-operator reliability. ICC values were evaluated using international standards, poor less than 0.40, moderate between 0.40 and 0.59, good between 0.60 and 0.74, and excellent between 0.75 and 1.00^17^. The standard error of measurement (SEM) was calculated using the formula SEM = SD × √("1 − ICC" ), and the MDC was calculated using MDC = 1.96 × SEM × √2.^14,18^. The coefficient of variance(CV = SD/mean × 100%) was computed. Intra- and inter-operator reliabilities are shown by the Bland and Altman graphs (Fig. 3). A paired t test was performed to compare the average shear modulus of TLF between 0° and 60° of upper body forward, and statistical significance was P < 0.05.

## Results

### Intra- and inter-operator reliabilities of TLF shear modulus

The ICC values of the intra-operator (ICC = 0.860–0.938) and inter-operator (ICC = 0.904–0.944) were excellent, SEM less than 1.7 kPa, MDC less than 4.71 kPa, and CV less than 6.3% (Table 1). Figure 3A is the Bland and Altman plot of intra-operator reliability at the L3, showing that the mean difference is 0.6 kPa and the 95% limits of agreement is − 3.6 to 4.8 kPa. Figure 3(B) is the Bland and Altman plot of inter-operator reliability at the L3, showing that the mean difference is 0.3 kPa and the 95% limits of agreement is − 6.0 to 6.6 kPa. Figure 3C is the Bland and Altman plot of intra-operator reliability at the L4, showing that the mean difference is − 0.1 kPa and the 95% limits of agreement is − 6.8 to 6.6 kPa. Figure 3D is the Bland and Altman plot of inter-operator reliability at the L4, showing that the mean difference is 0 kPa and the 95% limits of agreement is − 8.0 to 8.0 kPa.

**Table 1:** Intra- and inter-operator reliabilities of SWE for thoracolumbar fascia shear modulus.

	L3			L4		
	Mean ± SD (kPa)	SEM	MDC	Mean ± SD (kPa)	SEM	MDC
Operator A in test1	117.2 ± 6.3	1.4	3.88	136.9 ± 6.9	1.5	4.16
Operator A in test2	116.6 ± 5.9	1.3	3.60	137.0 ± 6.1	1.4	3.88
Operator B	116.9 ± 7.4	1.7	4.71	136.9 ± 6.6	1.5	4.16
ICC(intrao-perator)	0.938(0.851–0.975)			0.860(0.681–0.942)		
ICC(intero-perator)	0.944(0.859–0.978)			0.904(0.755–0.962)		

*SD* standard deviation (kPa); *SEM* standard error mean (kPa); *MDC* minimum detectable change (kPa); *kPa* kilo Pascal; *ICC* intraclass correlation coefficient; *95% CI* 95% confidence interval.

### Changes in the shear modulus of the TLF

**Figure 4:**
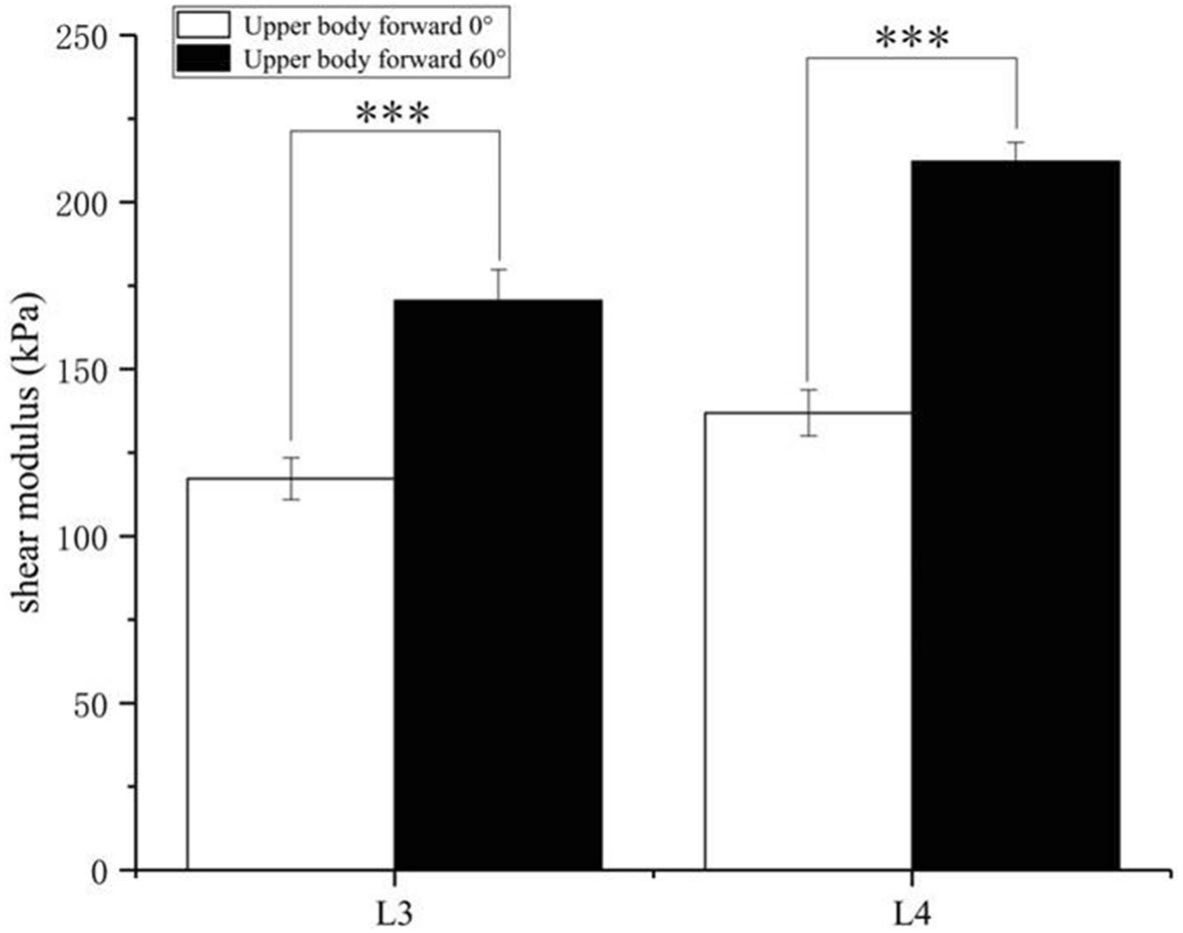
Mean and standard deviation of TLF shear modulus examined during 0° (white bar) or 60° (black bar) of upper body forward. ***Significant intergroup difference (P < 0.001).

At vertebral level L3, the shear modulus of the TLF at sitting-forward 60° (170.5 ± 9.3 kPa) was significant greater than that of the TLF at sitting forward 0° (117.2 ± 6.3 kPa) (*p* < 0.001. Figure 4). At vertebral level L4, the significant increase in the shear modulus of the TLF was found sitting-forward 60° (212.2 ± 5.7 kPa) compared to the sitting forward 0° (136.9 ± 6.9 kPa) (*p* < 0.001. Figure 4).

## Discussion

Overall, the present study results show that SWE is a reliable tool to quantify the dynamic change of TLF shear modulus. We found that the SWE has excellent intra- and inter-operator (ICC > 0.75) reliabilities. The relatively low values of SEM and MDC in the results prove the precision of the measurement. The stiffness of TLF increased significantly when sitting-forward 60° was compared with sitting-forward 0°.

This is the first study to examine the intra- and inter-operator reliabilities of elastic properties of TLF using SWE. Other studies of the same type only examined the reliability of measuring the stiffness of skeletal muscles using the SWE device. For example, Moreau et al.^16^ used SWE to quantify the shear modulus of multifidus muscle (L2–3, L4–5) with high reliability (ICC = 0.72–0.95). Blain et al.^17^ studied the reliability of SWE in measuring the shear modulus of multifidus and erector spinalis muscles in five different postures. The results showed that the reliability of SWE in various postures ranged from general to excellent (ICC = 0.386–0.862), which the SWE had the ability to quantify the dynamic changes of the measuring multifidus and erector spinalis muscles. But the shear modulus of TLF was not measured. Our results are similar to those studies for measuring skeletal muscle stiffness using the SWE device. In addition to using ICC values to assess reliability, our study also used the Bland–Altman plot provides visual evaluation for limits of agreement. As shown in Fig. 3, all data points were within the 95% consistency limit, which indicates that the intra- and inter-operator reliabilities have high consistency. Therefore, the Bland–Altman plots further verified the reliability of our experimental results.

Five days after the initial test, the same subject was retested by researcher A. The experimental results showed a high consistency, but intra-operator reliability (ICC = 0.860–0.938) was lower than inter-operator reliability (ICC = 0.904–0.944). Zhang et al.^14^ revealed similar results that the inter-operator reliability (ICC = 0.94–0.98) were higher than intra-operator reliability (ICC = 0.85–0.86) for quantifying the shear modulus of upper trapezius using the SWE. The possible explanation is that the amount of exercise and other uncontrollable factors during the 5 days may have influenced the reliability of repeated measurements.

In this experiment, we also calculated the MDC of sitting position, which reflects the precision of the device and real change. In terms of our research results, the measurements of TLF stiffness in other positions should be 4.71 kPa more than that in sitting position to reflect real change.

The present study revealed an increase of 45.5% (L3) or 55.0% (L4) in the shear modulus of the TLF from 0° to 60° of upper body forward tilt. The stiffness variation value from 0° to 60° is greater than the MDC (4.71 kPa) of sitting position, which indicates that the stiffness change from sitting-forward 60° was caused by real change rather than error. Because there are only a few studies that consider the shear modulus of TLF from the perspective of a healthy person, it is difficult to compare our results directly with the results of previous studies. However, previous studies investigated the passive tensile response of shear modulus of multifidus muscle similar with our results at the same location. For example, Moreau et al.^16^ used SWE to measure the shear modulus of the multifidus at L3, and they found that the shear modulus of the multifidus at passive stretching (13.8 ± 2.9 kPa) was greater than that at prone posture (8.5 ± 1.9 kPa).In addition, Langevin et al.^5^ found that the TLF shear strain in people with chronic lower back pain was 20% lower than that in healthy subjects. Therefore, the increase of TLF stiffness in the present experimental resultsfurther verifies that poor posture may be one of the potential factors causing LBP.

In our study, we observed that the elastic images showed uneven stiffness of TLF.This phenomenon may be related to the anatomical structure of TLF and the uneven distribution of tension. In terms of anatomical structure, the posterior layer of TLF is further divided into three thinner sub-layers (superficial, middle, deep). The superficial layer, the middle layer and the deep layer is regarded as the continuation of the deep fascia of latissimus dorsi, the continuation of the tendon of latissimus dorsi, and a loose connective tissue composed of longitudinal and transverse collagen fibers crisscross arrangement respectively^19^. This indicates that the TLF is a composite structure composed of fascial layer, ligament and loose connective tissue, which is heterogeneous in itself. In addition, the fiber of each layer in the three-layer structure of TLF has a specific direction, and the movement between each layer is relatively independent, so the response of TLF to the change of tension has strong anisotropy^5,19^. Therefore, we take three adjacent ROIs to expand the detection range to reduce the impact caused by the heterogeneity during the test. According to the reliability research results of the experiment, our method has beensuccessful.

There are some limitations to this study. First, this study recruited only young men as the research objects. In previous studies, the difference in age was a potential confounding factor, because aging affects the myofascial muscle tension by reducing the number of muscles fibers and their cross-sectional area^20^. Another potential factor was gender, and an increased in estrogen may lead to sagging of muscles and ligaments and thickening of TLF^21^. Hence, only healthy young men were selected in this study to rule out age- and gender-related problems; thus, the effect of age and gender differences on fascia stiffness cannot be assessed. We need to include females and subjects of different ages in future research. Second, the subjects were healthy people with no discomfort or pain in their back. Therefore, future studies should be focused on the biomechanical changes of TLF in people with low back discomfort, to explore the pathogenesis of LBP and improve the clinical applicability of SWE.

## Conclusions

The study demonstrated the SWE is a reliable tool to quantify the stiffness of TLF, and the stiffness change more than 4.71 kPa can be considered as a true change rather than error. Furthermore, this technique is capable ofdetecting the change of TLF between sitting forward 0° and sitting-forward 60°, which provides the possibility for further studies the dynamic changes of TLF.
